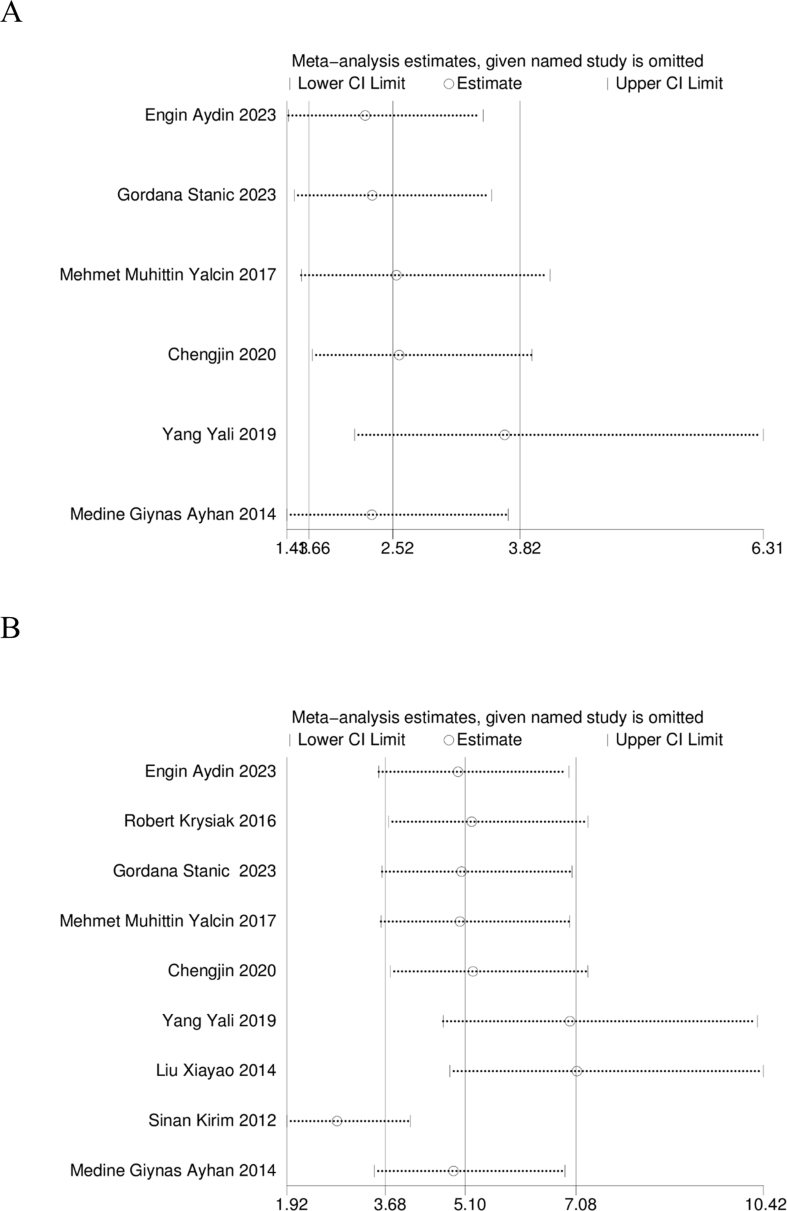# Association between depression and anxiety disorders with euthyroid Hashimoto's thyroiditis: A systematic review and meta-analysis

**DOI:** 10.1016/j.cpnec.2024.100279

**Published:** 2024-12-02

**Authors:** Bo Wang, Jie Huang, Li Chen

**Affiliations:** a Department of Paediatric Surgery, Tianjin Medical University General Hospital Tianjin, China; b Department of Respiratory and Critical Care Medicine, Tianjin Medical University General Hospital, Tianjin, China; c Department of Medicine IV, LMU University Hospital, LMU Munich, Munich, Germany

**Keywords:** Hashimoto's thyroiditis, Anxiety disorder, Depression, Meta-analysis

## Abstract

**Background:**

Hashimoto's thyroiditis (HT) affects up to 10 % of the population and is a common cause of hypothyroidism, which can lead to depression and anxiety. However, it remains unclear whether HT directly causes these conditions or if they arise due to HT-induced hypothyroidism. The present review aims to offer meta-analytic insights into the relationship between depression and anxiety in patients with euthyroid HT.

**Methods:**

A comprehensive search was conducted in PubMed, Embase, Web of Science, Cochrane Library, CNKI, Wanfang Data, SinoMed, and VIP from their inception through May 2024. Case-control or cross-sectional studies examining the association between euthyroid HT and either depression, anxiety disorders, or both were included.

**Results:**

For depression, 1365 patients (694 HT vs. 671 controls) from 11 articles were analyzed; for anxiety, 1009 patients (516 HT vs. 493 controls) from 8 articles were included. HT patients had 2.5 times higher odds of anxiety disorders (OR = 2.52, 95 % CI: 1.66–3.82). The Beck Depression Inventory showed a WMD of 4.26 (95 % CI: 1.28–7.24) and the Beck Anxiety Inventory a WMD of 5.10 (95 % CI: 1.55–8.66).

**Limitation:**

The findings should be interpreted cautiously due to heterogeneity, potential publication bias, and variability in assessment tools, which may limit generalizability.

**Conclusions:**

Euthyroid HT patients exhibit a higher prevalence of anxiety disorders compared to healthy control groups, and more susceptible to anxiety and depression symptoms based on the Beck Inventory. Thyroid antibodies themselves are also associated with depression and anxiety disorder.

## Highlights

• Euthyroid HT linked to higher odds of anxiety disorders (OR = 2.52).
• Beck Depression Inventory shows higher scores in HT patients (WMD = 4.26).
• HT patients more susceptible to anxiety (WMD = 5.10) based on Beck Inventory.
• Thyroid antibodies associated with depression and anxiety symptoms.

## 1 Introduction

Hashimoto's thyroiditis (HT) is an autoimmune disorder characterized by T cell infiltration and progressive destruction of thyroid tissue, leading to hypothyroidism [[1], [2], [3]], with diagnosis typically involving the detection of thyroid-specific antibodies and diffuse changes in the thyroid gland observed through ultrasonography [4,5]. In parallel, depressive and anxiety disorders are prevalent mental health conditions [6], whose incidence has been further heightened by the global COVID-19 pandemic [7]. Emerging evidence points to a potential link between HT and an increased risk of depressive and anxiety disorders [8], a relationship largely attributed to hypothyroidism, as thyroid hormone deficiencies often result in emotional disturbances such as depression and anxiety [[9], [10], [11], [12], [13], [14], [15]].

Interestingly, recent studies have proposed that thyroid antibodies may be independently linked to depression and anxiety disorders, regardless of thyroid function [8,16]. This hypothesis is supported by clinical studies in euthyroid patients [[17], [18], [19], [20]], as well as neuroimaging research [21]and animal studies involving euthyroid mice [22], all of which have reported similar findings. Nevertheless, some studies hold a differing view, finding no direct association between depression and thyroid antibodies [23]. Similarly, the relationship between thyroid antibodies and anxiety disorders remains contentious, with conflicting results across the literature [24,25]. Therefore, the role of thyroid antibodies in the development of depression and anxiety remains a subject of ongoing debate.

Moreover, a recent meta-analysis has indicated that patients with HT have an elevated risk of developing depression and anxiety disorders [8]. However, this analysis did not specifically compare euthyroid HT patients with healthy controls. Given that depression and anxiety are significant global public health concerns, each affecting approximately 300 million people worldwide [26], it becomes increasingly crucial to further investigate their underlying causes. Accordingly, our meta-analysis aims to systematically evaluate the available literature to determine whether euthyroid HT patients are at an increased risk of anxiety and depression compared to healthy controls.

## 2 Methods

### 2.1 Search strategy

This meta-analysis adhered to the PRISMA (Preferred Reporting Items for Systematic Reviews and Meta-Analysis) 2020 guidelines [27] and was prospectively registered in PROSPERO (CRD42024545078), as detailed in Supplementary Table S1. The literature search was conducted from inception until May 2024, across PubMed, Embase, Web of Science, Cochrane Library, CNKI, Wanfang Data, SinoMed, and VIP, and included abstracts in English and Chinese. Search terms included "Hashimoto Disease", "Autoimmune Thyroiditis", "Depression", and "Anxiety" as specified in Supplementary Table S2.

### 2.2 Selection criteria

Studies were included based on the following criteria: 1) Study Design: Only case-control or cross-sectional studies. 2) Participants: Euthyroid Hashimoto's thyroiditis (HT) patients and healthy controls. 3) Outcomes: Studies assessing and comparing anxiety or/and depression. 4) Data Reporting: Availability of data to calculate odds ratios (OR), weighted mean differences (WMD), or standardized mean differences (SMD). Excluded document types included reviews, letters, editorial comments, and case reports. Studies lacking a control group comparison were also omitted.

### 2.3 Recorded variables

Data extraction was performed independently by two researchers, with a third investigator intervening to resolve disputes and make the final decisions. Baseline patient information, including both categorical and continuous variables, was collected, along with outcome variables such as the number of patients and their depression or anxiety assessment scores. For continuous variables presented as medians with ranges or interquartile ranges, a validated online tool was used to convert these values into mean ± standard deviation (SD). This tool is available at https://www.math.hkbu.edu.hk/∼tongt/papers/median2mean.html. Study quality was assessed using the Newcastle–Ottawa Scale (NOS), which evaluates selection, comparability, and exposure across eight criteria. Studies with a score between seven and nine were considered high quality.

### 2.4 Statistical analysis

Review Manager 5.3 software (Cochrane Collaboration, Oxford, UK) were utilized for our meta-analysis. For continuous and binary outcomes, WMD and SMD, along with OR were reported, each reported with 95 % confidence intervals (CIs). The choice between WMD and SMD was determined by the uniformity of measurement scales across studies for the same intervention. Significant heterogeneity was defined by a chi-squared test P value < 0.05 or an I^2 statistic >50 %. Depending on the presence of heterogeneity, a random-effects model or a fixed-effects model either applied. For the assessment of potential publication bias, funnel plots were generated using Review Manager 5.3, and Egger's regression tests were conducted using Stata 12.0 (Stata Corp, College Station, TX, USA). In addition, one-way sensitivity analyses were conducted to assess the impact of individual studies on the aggregate outcomes for those exhibiting significant heterogeneity. P < 0.05 was regarded as indicative of significant publication bias.

## 3 Results

### 3.1 Database

The flowchart of the search and selection process is illustrated in Fig. 1. Among the 11 articles included, all specifically discuss depression, with 8 of these also addressing anxiety (See Table 1). For the pooled analysis, the studies on depression included data from 1365 patients (694 HT vs. 671 Normal Controls). Similarly, the studies on anxiety encompassed data from 1009 patients (516 HT vs. 493 Normal Controls). Basic information on the included articles is provided in Table S1. Additionally, Supplementary Table S3 summarizes the demographic and clinical characteristics, along with the NOS scores, of the selected studies.

**Fig. 1:**
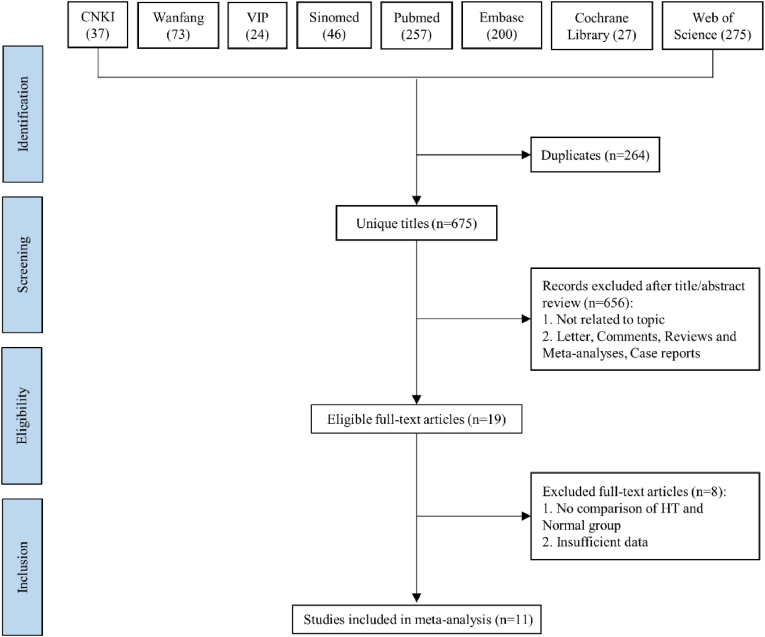
Flowchart of the systematic search and selection process.

**Table 1:** Baseline characteristics and methodological assessment of included studies.

Authors	Country	Study design	Study period	Patients (n) HT/Normal	NOS score
Mehmet Muhittin Yalcin [17]	Turkey	Cross-sectional study	–	93/31	8
Medine Giynas Ayhan [18]	Turkey	Cross-sectional study	–	51/68	8
Gordana Stanić [19]	Serbia	Case-control study	2021.8–2022.2	24/73	8
Sinan Kırım [20]	Turkey	Cross-sectional study	–	94/107	6
Engin Aydin [28]	Turkey	Case-control study	–	41/41	7
Robert Krysiak [29]	Poland	Cross-sectional study	–	16/18	7
Olga Vasovic [30]	Serbia	Cross-sectional study	–	130/111	7
Chengjin [31]	China	Case-control study	2018.1–2019.5	47/40	7
Yang Yali [32]	China	Case-control study	2014.8–2016.2	89/94	8
Liu Xiayao [33]	China	Case-control study	2013.1–2014.2	68/53	8
Georg Zettinig [34]	Austria	Cross-sectional study	–	41/38	6

### 3.2 Meta-analytic association between HT and depression

A total of 11 studies provided outcome data for HT and depression. Depression was primarily evaluated using self-report questionnaires. Specifically, 36 % of the studies (4 samples) utilized versions of the Beck Depression Inventory, 18 % (2 samples) employed the Hamilton Depression Scale, another 18 % (2 samples) employed the Zung's Self-Rating Depression Scale, and other studies used the Hospital Depression Scale and the Four-Dimensional Symptom Questionnaire (4DSQ), among others. Psychiatric disorders were consistently defined through established cutoff values on these dimensional scales.

The analysis revealed a significantly higher likelihood of depressive symptoms in HT patients compared to healthy controls. The Beck Depression Inventory indicated a Weighted Mean Difference (WMD) of 4.26 (95 % CI: 1.28–7.24), suggesting that individuals with HT are approximately 4.3 points higher on the depression scale compared to healthy controls (Fig. 2B). This finding was accompanied by high heterogeneity (I^2^ = 89 %, p < 0.00001) (Fig. S1B). However, Egger's test did not show statistical significance for publication bias (p = 0.152).

**Fig. 2:**
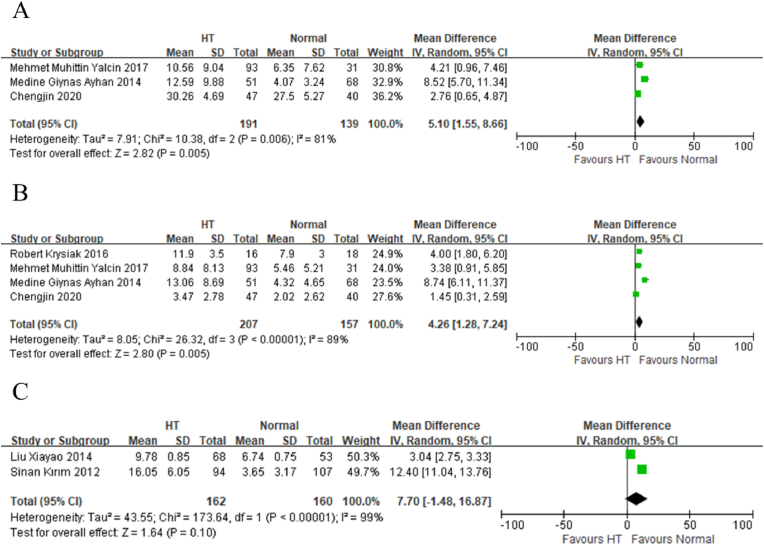
Forest plots of assessment tools: (A) Beck Anxiety Inventory (B) Beck Depression Inventory (C) Hamilton Depression Scale.

Similarly, the Hamilton Depression Scale demonstrated an increased likelihood of depressive symptoms in the HT cohort, with a WMD of 7.70 (95 % CI: −1.48-16.87) (Fig. 2C). This analysis also exhibited statistically significant heterogeneity (I^2^ = 99 %, p < 0.00001) (Fig. S1C).

**Fig. 3:**
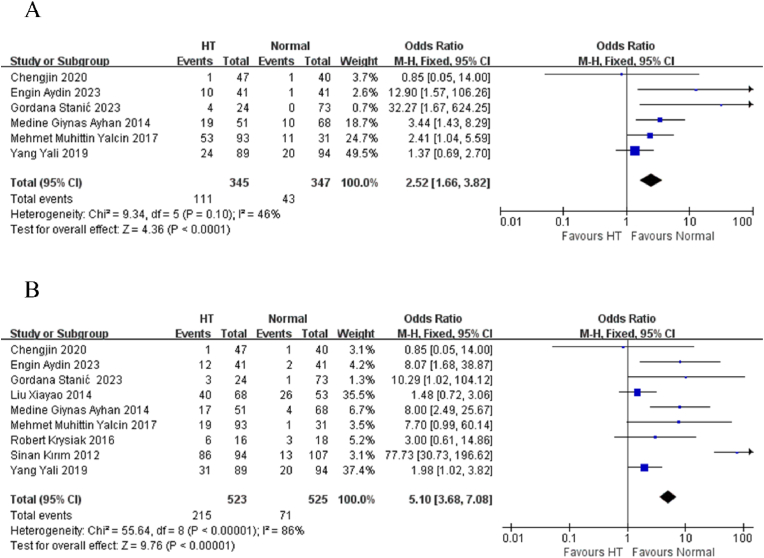
Forest plots of prevalence: (A) Anxiety (B) Depression.

In terms of prevalence, we found that the likelihood of developing depression is 3.5 times higher among patients with HT compared to healthy controls (OR = 5.10, 95 % CI: 3.68–7.08) (Fig. 3B). This analysis showed significant heterogeneity (I^2^ = 86 %, p < 0.00001) and visual evidence of publication bias (Fig. S1D). Additionally, Egger's test confirmed the presence of publication bias (p = 0.002). Unfortunately, the two studies using Zung's Self-Rating Depression Scale cannot be analyzed together, as one only reports the prevalence rate, while the other provides only the scores.

### 3.3 Meta-analytic association between HT and anxiety disorders

There are 8 studies related to HT and anxiety disorders. 38 % of the studies (3 samples) employing versions of the Beck Anxiety Inventory, 2 studies utilized tools Zung Self-Rating Anxiety Scale, while other used such as the Hospital Anxiety Scale. etc. The analysis revealed a significantly elevated likelihood of anxiety symptoms in HT patients compared to healthy controls. Specifically, the Beck Anxiety Inventory indicated a WMD of 5.10 (95 % CI: 1.55–8.66), suggesting that individuals with HT are approximately 5.1 times more likely to exhibit anxiety symptoms (Fig. 2A). This finding was characterized by high heterogeneity (I^2^ = 81 %, p = 0.006) (Fig. S1A). However, Egger's test did not indicate statistical significance for publication bias (p = 0.625).

Regarding prevalence, the odds of developing an anxiety disorder were found to be 2.5 times higher in HT patients compared to healthy controls (OR = 2.52, 95 % CI: 1.66–3.82) (Fig. 3A). This result showed no significant heterogeneity (I^2^ = 46 %, p = 0.10) and neither statistical (Egger's test, p = 0.402) nor visual (Fig. S1E) evidence of publication bias.

### 3.4 Sensitivity analysis

We conducted one-way sensitivity analyses to compare the Beck Anxiety Inventory Score, Beck Depression Inventory Score, and the prevalence of Anxiety and Depression. By systematically excluding each study, we assessed the impact of individual studies on the overall WMD or OR. The sensitivity analyses revealed that the overall effect size remained stable regardless of the exclusion of any single study for the Beck Anxiety Inventory Score (Fig. S2A), Beck Depression Inventory Score (Fig. S2B), prevalence of Anxiety (Fig. S3A), and prevalence of Depression (Fig. S3B). When we excluded the data reported by Chengjin in 2020 [31], the heterogeneity in the Beck Anxiety Inventory Score disappeared (I2 = 74 %, p = 0.05), indicating that this study accounts for most of the observed heterogeneity. Similarly, excluding the data reported by Medine [18] eliminated the heterogeneity in the Beck Depression Inventory Score (I2 = 61 %, p = 0.08), suggesting that these studies are major contributors to the heterogeneity. Furthermore, the exclusion of data reported by Sinan Kirim in 2012 [20] resulted in the disappearance of heterogeneity in the Prevalence of Depression (I^2^ = 40 %, p = 0.11), highlighting this study's significant impact on the overall heterogeneity.

## 4 Discussion

Hashimoto's thyroiditis (HT) has seen a marked increase in prevalence over recent years, particularly in Europe, where the rate has reached approximately 7.8 % [35]. Simultaneously, depressive and anxiety disorders—two of the most common psychiatric conditions—have also surged globally [7,36]. Notably, the COVID-19 pandemic exacerbated this trend, leading to a 27.6 % rise in cases of major depressive disorder and a 25.6 % increase in anxiety disorder cases worldwide [37]. Although numerous studies have explored the potential causal relationship between HT and these psychiatric disorders, the association remains contentious and inconclusive.

Given the critical role thyroid hormones play in the central nervous system— impacting the production of neurotrophic factors, learning and memory mechanisms, and the regulation of brain stem cell fate [[38], [39], [40], [41], [42], [43], [44]], as well as mood regulation [12]—the association between HT and psychiatric disorders is frequently attributed to hypothyroidism induced by HT [45,46]. This connection dates back to 1825 when Parry observed heightened "nerve strokes" associated with thyroid disease. In 1873, Seagull identified a link between myxedema and psychosis. The term "myxedema madness" coined by Asher in 1949, was introduced to describe the psychiatric disturbances seen in patients with hypothyroidism [[47], [48], [49]]. Numerous studies have since confirmed the association between hypothyroidism and depression [9,50,51].

In addition to the psychiatric disorders attributed to hypothyroidism in HT, emerging evidence suggests that thyroid antibodies may be independently associated with depression and anxiety disorders, irrespective of thyroid function. This hypothesis is supported by findings from research on Hashimoto's encephalopathy, where autoantibodies have been shown to exert significant effects on the central nervous system [52]. Further, the detection of anti-thyroid peroxidase and anti-thyroglobulin autoantibodies in the cerebrospinal fluid of patients with unipolar depression reinforces the potential role of immune mechanisms in psychiatric conditions beyond thyroid dysfunction [53]. However, some studies have challenged the role of thyroid antibodies in anxiety and depression, raising questions about their independent influence [23,25]. Notably, HT can manifest across a spectrum of thyroid states, ranging from overt hypothyroidism to subclinical hypothyroidism, and even in euthyroid individuals [[17], [18], [19], [20],[28], [29], [30], [31], [32], [33]]. This diversity of clinical presentations offers valuable opportunities for further research into the complex interactions between HT and psychiatric disorders.

Therefore, we present what we believe to be the first meta-analysis to systematically review anxiety and depression in euthyroid patients with HT. This review initially included 11 articles, all of which were related to depression, and 8 of which also addressed anxiety. However, due to the absence of patient data from Olga Vasovic [30]'s study, the prevalence calculations were based on 9 articles for depression and 6 articles for anxiety. Our robust findings support the hypothesis that euthyroid patients with HT exhibit a higher prevalence of anxiety disorder compared to healthy control groups. The forest plots for the Beck Anxiety Inventory and Beck Depression Inventory scores show significant heterogeneity, with I^2^ values of 81 % and 89 %, respectively. However, Egger's test results for both measures were greater than 0.05, indicating no significant publication bias. Despite the heterogeneity, the absence of publication bias supports the robustness of our meta-analysis results. Thus, we can conclude that euthyroid HT patients are more susceptible to anxiety and depression symptoms compared to healthy individuals based on the Beck Inventory.

Some animal studies and imaging evidence also support our findings. Yao-Jun Cai et al. [22]. discovered that in an euthyroid HT mouse model, mice exhibited more anxiety-like behavior in open field and elevated plus maze tests, and more depressive-like behavior in forced swimming and tail suspension tests compared to controls. M. Piga et al. [21]. found a high prevalence of brain perfusion abnormalities in euthyroid HT, similar to those observed in severe Hashimoto's encephalopathy.

Our analysis includes a rigorous and comprehensive literature review, delivering high-quality data and generating outcome measures that remain robust against the studies' inherent risk of bias. However, several limitations should be acknowledged. The substantial heterogeneity observed in the prevalence of depression and potential publication bias may have led to a slight overestimation of effect size. Moreover, considerable variability was detected in the statistical outcomes of continuous variables, such as the Beck Anxiety Inventory and Beck Depression Inventory scores. The studies included in this meta-analysis employed different assessment tools and covered relatively short follow-up periods, which may introduce biases and limit the generalizability of the findings. Consequently, the results should be interpreted with caution, particularly in light of potential confounding factors. Future research should focus on well-designed case-control studies with longer follow-up periods to further clarify the comparative levels of anxiety and depression in patients with euthyroid Hashimoto's thyroiditis.

## 5 Conclusion

In conclusion, pooled analyses demonstrated that euthyroid HT patients exhibit a higher prevalence of anxiety disorders compared to healthy control groups. Based on the Beck Inventory, euthyroid HT patients are more susceptible to anxiety and depression symptoms than healthy individuals. We believe that thyroid antibodies themselves are also associated with depression and anxiety disorder based on our research.

## CRediT authorship contribution statement

**Bo Wang:** Visualization, Methodology, Data curation. **Jie Huang:** Visualization, Validation. **Li Chen:** Writing – review & editing, Writing – original draft.

## Funding sources

No fundings support.

## Declaration of competing interest

All authors declare that they have no conflicts of interest.

## Figures and Tables

**figs1:**
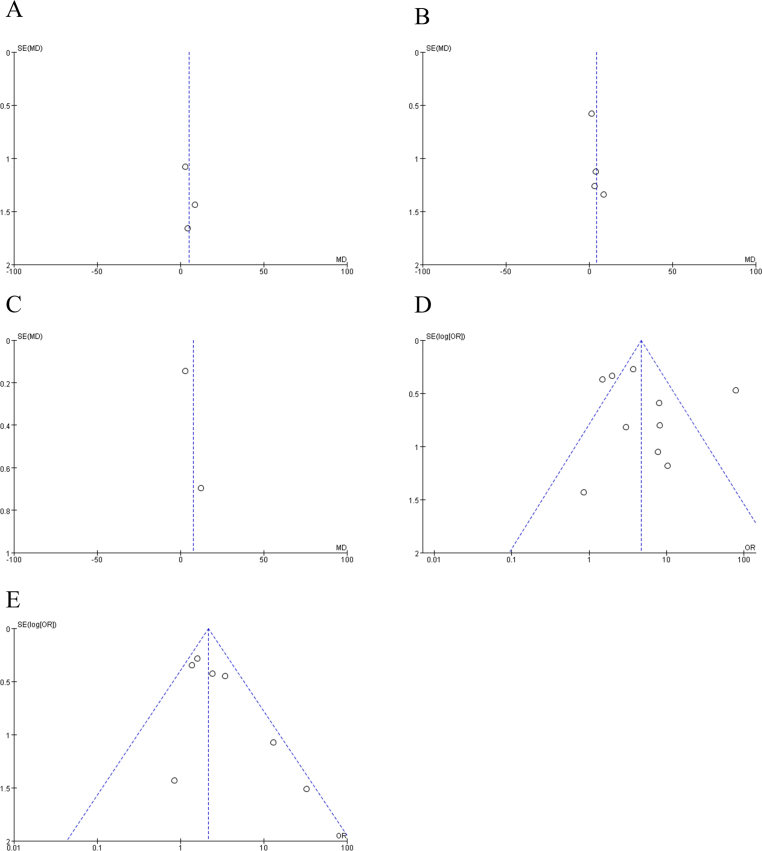


**figs2:**
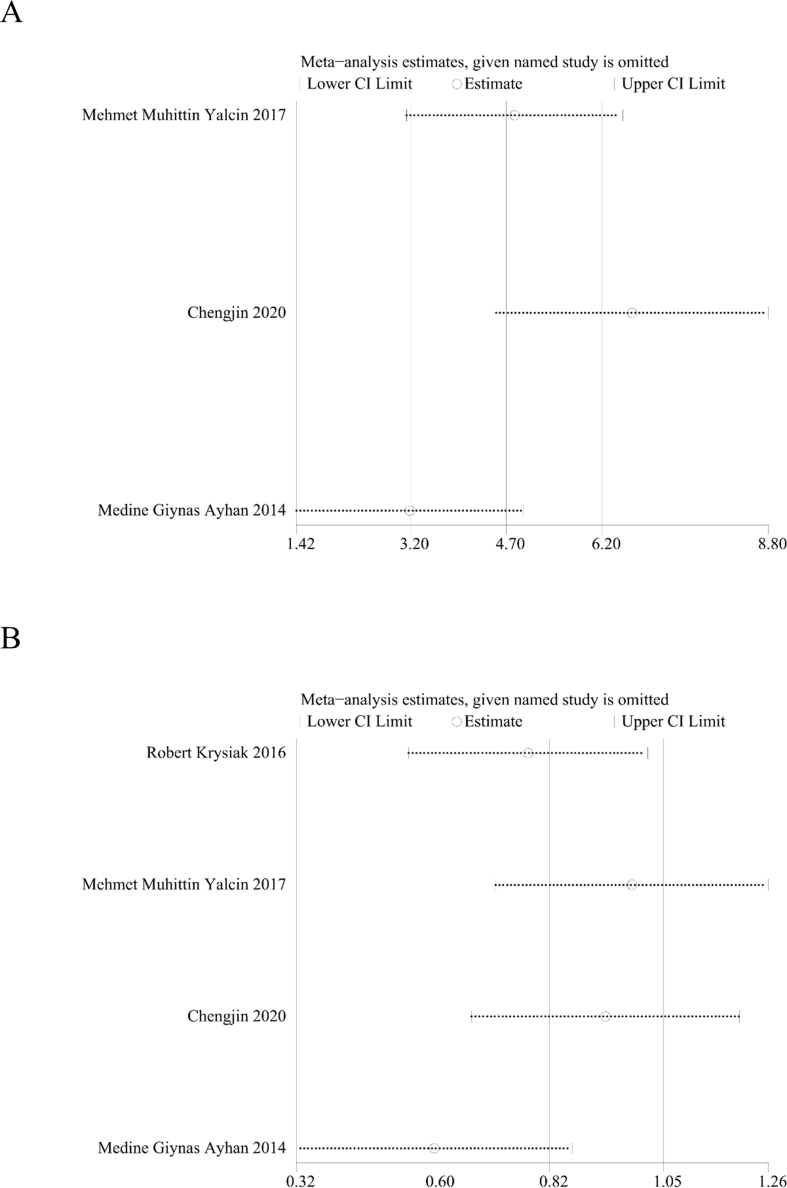


**figs3:**