# Supplementary material for: Association between depression and anxiety disorders with euthyroid Hashimoto's thyroiditis: A systematic review and meta-analysis
Source: Compr Psychoneuroendocrinol. 2024 Dec 2;20:100279. doi: 10.1016/j.cpnec.2024.100279 (PMC11665666; doi:10.1016/j.cpnec.2024.100279)
Supplement: Multimedia component 2 [file mmc2.docx]

# Supplementary Table 2 Systematic search detail

| **The search strategy for PubMed** | | |
| --- | --- | --- |
| **Sequence** | **Search terms** | **Hits** |
| #1 | ((((((((((((((((((((((((((((((((((Hashimoto Disease[MeSH Terms]) OR (Disease, Hashimoto[Title/Abstract])) OR (Chronic Lymphocytic Thyroiditis[Title/Abstract])) OR (Chronic Lymphocytic Thyroiditides[Title/Abstract])) OR (Lymphocytic Thyroiditides, Chronic[Title/Abstract])) OR (Lymphocytic Thyroiditis, Chronic[Title/Abstract])) OR (Thyroiditides, Chronic Lymphocytic[Title/Abstract])) OR (Thyroiditis, Chronic Lymphocytic[Title/Abstract])) OR (Hashimoto Struma[Title/Abstract])) OR (Hashimoto's Struma[Title/Abstract])) OR (Hashimoto's Syndrome[Title/Abstract])) OR (Hashimoto Syndrome[Title/Abstract])) OR (Hashimoto's Syndromes[Title/Abstract])) OR (Hashimotos Syndrome[Title/Abstract])) OR (Syndrome, Hashimoto's[Title/Abstract])) OR (Syndromes, Hashimoto's[Title/Abstract])) OR (Hashimoto's Disease[Title/Abstract])) OR (Disease, Hashimoto's[Title/Abstract])) OR (Hashimotos Disease[Title/Abstract])) OR (Hashimoto Thyroiditis[Title/Abstract])) OR (Hashimoto Thyroiditides[Title/Abstract])) OR (Thyroiditides, Hashimoto[Title/Abstract])) OR (Thyroiditis, Hashimoto[Title/Abstract])) OR (Thyroiditis, Autoimmune[Title/Abstract])) OR (Autoimmune Thyroiditides[Title/Abstract])) OR (Thyroiditides, Autoimmune[Title/Abstract])) OR (Autoimmune Thyroiditis[Title/Abstract])) OR (Thyroiditis, Lymphocytic[Title/Abstract])) OR (Lymphocytic Thyroiditides[Title/Abstract])) OR (Lymphocytic Thyroiditis[Title/Abstract])) OR (Thyroiditides, Lymphocytic[Title/Abstract])) OR (Thyroiditis, Lymphomatous[Title/Abstract])) OR (Lymphomatous Thyroiditides[Title/Abstract])) OR (Lymphomatous Thyroiditis[Title/Abstract])) OR (Thyroiditides, Lymphomatous[Title/Abstract]) | 24,886 |
| #2 | ((((((((((((((Depression[MeSH Terms]) OR (Depressive Symptoms[Title/Abstract])) OR (Depressive Symptom[Title/Abstract])) OR (Symptom, Depressive[Title/Abstract])) OR (Emotional Depression[Title/Abstract])) OR (Depression, Emotional[Title/Abstract])) OR (Anxiety[Title/Abstract])) OR (Angst[Title/Abstract])) OR (Social Anxiety[Title/Abstract])) OR (Anxieties, Social[Title/Abstract])) OR (Anxiety, Social[Title/Abstract])) OR (Social Anxieties[Title/Abstract])) OR (Hypervigilance[Title/Abstract])) OR (Nervousness[Title/Abstract])) OR (Anxiousness[Title/Abstract]) | 512,034 |
| #3 | #1 AND #2 | 257 |
| **The search strategy for Web of science** | | |
| **Sequence** | **Search terms** | **Hits** |
| #1 | Hashimoto Disease (Topic) or Disease, Hashimoto (Topic) or Chronic  Lymphocytic Thyroiditis (Topic) or Chronic Lymphocytic Thyroiditides  (Topic) or Lymphocytic Thyroiditides, Chronic (Topic) or Lymphocytic  Thyroiditis, Chronic (Topic) or Thyroiditides, Chronic Lymphocytic (Topic)  or Thyroiditis, Chronic Lymphocytic (Topic) or Hashimoto Struma (Topic)  or Hashimoto's Struma (Topic) or Hashimoto's Syndrome (Topic) or  Hashimoto Syndrome (Topic) or Hashimoto's Syndromes (Topic) or  Hashimotos Syndrome (Topic) or Syndrome, Hashimoto's (Topic) or  Syndromes, Hashimoto's (Topic) or Hashimoto's Disease (Topic) or Disease,  Hashimoto's (Topic) or Hashimotos Disease (Topic) or Hashimoto Thyroiditis  (Topic) or Hashimoto Thyroiditides (Topic) or Thyroiditides, Hashimoto  (Topic) or Thyroiditis, Hashimoto (Topic) or Thyroiditis, Autoimmune  (Topic) or Autoimmune Thyroiditides (Topic) or Thyroiditides, Autoimmune  (Topic) or Autoimmune Thyroiditis (Topic) or Thyroiditis, Lymphocytic  (Topic) or Lymphocytic Thyroiditides (Topic) or Lymphocytic Thyroiditis  (Topic) or Thyroiditides, Lymphocytic (Topic) or Thyroiditis,  Lymphomatous (Topic) or Lymphomatous Thyroiditides (Topic) or Lymphomatous Thyroiditis (Topic) or Thyroiditides, Lymphomatous (Topic) | 14,636 |
| #2 | **Depression (Topic) or Depressive Symptoms (Topic) or Depressive Symptom (Topic) or Symptom, Depressive (Topic) or Emotional Depression (Topic) or Depression, Emotional (Topic) or Anxiety (Topic) or Angst (Topic) or Social Anxiety (Topic) or Anxieties, Social (Topic) or Anxiety, Social (Topic) or Social Anxieties (Topic) or Hypervigilance (Topic) or Nervousness (Topic) or Anxiousness (Topic)** | 981,718 |
| #3 | #1 AND #2 | 275 |
| **The search strategy for Cochrane Library** | | |
| **Sequence** | **Search terms** | **Hits** |
| #1 | MeSH descriptor: [Hashimoto Disease] explode all trees | 99 |
| #2 | (Chronic Lymphocytic Thyroiditis):ti,ab,kw OR (Hashimoto's Syndrome):ti,ab,kw OR (Thyroiditides, Hashimoto):ti,ab,kw OR (Syndromes, Hashimoto's):ti,ab,kw OR (Hashimotos Disease):ti,ab,kw | 65 |
| #3 | (Disease, Hashimoto):ti,ab,kw OR (Hashimoto's Disease):ti,ab,kw OR (Syndrome, Hashimoto's):ti,ab,kw OR (Thyroiditis, Hashimoto):ti,ab,kw OR (Hashimoto's Syndromes):ti,ab,kw | 302 |
| #4 | (Lymphocytic Thyroiditis, Chronic):ti,ab,kw OR (Hashimotos Syndrome):ti,ab,kw OR (Thyroiditis, Chronic Lymphocytic):ti,ab,kw OR (Hashimoto Thyroiditis):ti,ab,kw OR (Thyroiditides, Chronic Lymphocytic):ti,ab,kw | 251 |
| #5 | (Hashimoto Thyroiditides):ti,ab,kw OR (Hashimoto Struma):ti,ab,kw OR (Hashimoto's Struma):ti,ab,kw OR (Hashimoto Syndrome):ti,ab,kw OR (Chronic Lymphocytic Thyroiditides):ti,ab,kw | 27 |
| #6 | (Disease, Hashimoto's):ti,ab,kw OR (Lymphocytic Thyroiditides, Chronic):ti,ab,kw OR (Hashimoto Disease):ti,ab,kw | 241 |
| #7 | #1 or #2 or #3 or #4 or #5 or #6 | 307 |
| #8 | MeSH descriptor: [Thyroiditis, Autoimmune] explode all trees | 183 |
| #9 | (Thyroiditis, Autoimmune):ti,ab,kw OR (Thyroiditides, Lymphomatous):ti,ab,kw OR (Lymphomatous Thyroiditis):ti,ab,kw OR (Thyroiditis, Lymphomatous):ti,ab,kw OR (Autoimmune Thyroiditis):ti,ab,kw | 310 |
| #10 | (Lymphomatous Thyroiditides):ti,ab,kw OR (Thyroiditides, Lymphocytic):ti,ab,kw OR (Lymphocytic Thyroiditides):ti,ab,kw OR (Thyroiditides, Autoimmune):ti,ab,kw OR (Autoimmune Thyroiditides):ti,ab,kw | 0 |
| #11 | (Lymphocytic Thyroiditis):ti,ab,kw OR (Thyroiditis, Lymphocytic):ti,ab,kw | 26 |
| #12 | #8 or #9 or #10 or #11 | 377 |
| #13 | MeSH descriptor: [Depression] explode all trees | 18,240 |
| #14 | (Depression):ti,ab,kw OR (Depressive Symptoms):ti,ab,kw OR (Symptom,  Depressive):ti,ab,kw OR (Depressive Symptom):ti,ab,kw OR (Depression,  Emotional):ti,ab,kw | 108,045 |
| #15 | (Emotional Depression):ti,ab,kw | 8,308 |
| #16 | #13 or #14 or #15 | 108,045 |
| #17 | MeSH descriptor: [Anxiety] explode all trees | 12,604 |
| #18 | (Anxiety):ti,ab,kw OR (Social Anxiety):ti,ab,kw OR (Social  Anxieties):ti,ab,kw OR (Anxiety, Social):ti,ab,kw OR (Anxieties,  Social):ti,ab,kw | 77,848 |
| #19 | (Hypervigilance):ti,ab,kw OR (Nervousness):ti,ab,kw OR (Angst):ti,ab,kw  OR (Anxiousness):ti,ab,kw | 1,201 |
| #20 | #17 or #18 or #19 | 78,792 |
| #21 | #7 or #12 | 478 |
| #22 | #16 or #20 | 144,831 |
| #23 | #21 and #22 | 27 |
| **The search strategy for EMBASE** | | |
| **Sequence** | **Search terms** | **Hits** |
| #1 | (Hashimoto Disease or chronic lymphocytic thyroiditis or Hashimoto  autoimmune thyroiditis or Hashimoto goiter or Hashimoto thyroid disease or  Hashimoto thyroidism or Hashimoto thyroiditis or Hashimoto thyroidosis or  Hashimoto's disease or Hashimoto's thyroid disease or Hashimoto's  thyroidism or Hashimoto's thyroiditis or lymphadenoid goiter or lymphocytic  thyroiditis, chronic or struma hashimoto or struma lymphomatosa or Thyroiditis, Autoimmune or allergic thyroiditis or auto-immune thyroid disease or auto-immune thyroid disorder or auto-immune thyroiditis or autoimmune thyroid disease or autoimmune thyroid disorder or immune thyroiditis or lymphocytic thyroiditis or thyroid auto-immune disease or thyroid autoimmune disease or thyroiditis, autoimmune).ab. | 13,083 |
| #2 | (Depression or central depression or clinical depression or depressive disease or depressive disorder or depressive episode or depressive illness or depressive personality disorder or depressive state or depressive symptom or depressive syndrome or mental depression or parental depression or anticipatory anxiety or dental anxiety or eco-anxiety or fear of childbirth or fear of death or fear of falling or fear of missing out or fear of pain or math anxiety or performance anxiety or school anxiety or social anxiety or test anxiety).ab. | 582,493 |
| #3 | #1 AND #2 | 200 |
| **The search strategy for CNKI** | | |
| **Sequence** | **Search terms** | **Hits** |
| #1 | (SU %= 'Hashimoto's thyroiditis') OR (SU %= 'Hashimoto thyroiditis') OR (SU %= 'Hashimoto's disease') OR (SU %= 'Hashimoto disease') OR (SU %= 'autoimmune thyroiditis') OR (SU %= 'chronic lymphocytic thyroiditis') | 7,298 |
| #2 | (SU %= 'depression') OR (SU %= 'depressive disorder') OR (SU %= 'anxiety  disorder') OR (SU %= 'anxiety') | - |
| #3 | #2 is retrieved in the result of #1 | 37 |
| **The search strategy for Wanfang Data** | | |
| **Sequence** | **Search terms** | **Hits** |
| #1 | (Theme=Hashimoto's thyroiditis) OR (Theme=Hashimoto thyroiditis) OR (Theme=Chronic lymphocytic thyroiditis) OR (Theme=Autoimmune thyroiditis) OR (Theme=Hashimoto's disease) OR (Theme=Hashimoto disease) AND (Theme= depression) OR (Theme= depressive disorder) OR (Theme= anxiety disorder) OR (Theme= anxiety) | 73 |
| **The search strategy for SinoMed** | | |
| **Sequence** | **Search terms** | **Hits** |
| #1 | " Hashimoto's thyroiditis "[Common fields: Auto] OR " Hashimoto thyroiditis "[Common fields: Auto] OR " Chronic lymphocytic thyroiditis "[Common fields: Auto] OR " Autoimmune thyroiditis "[Common fields: Auto] OR " Hashimoto's disease "[Common fields: Auto] OR " Hashimoto disease "[Common fields: Auto] | 7,479 |
| #2 | " depression"[Common fields: Auto] OR " depressive disorder "[Common fields: Auto] OR " anxiety disorder "[Common fields: Auto] OR " anxiety "[Common fields: Auto] | 330,634 |
| #3 | #1 AND #2 | 46 |
| **The search strategy for VIP** | | |
| **Sequence** | **Search terms** | **Hits** |
| #1 | ((((((((((Title or Keywords = chronic lymphadenoid thyroiditis OR Title or Keywords = chronic lymphocyte thyroiditis) OR Title or Keywords = chronic lymphocytic thyroiditis) OR Title or Keywords = chronic lymphocytic thyroiditis) OR Title or Keywords = Hashimoto's disease) OR Title or Keywords = Hashimoto's thyroiditis) OR Title or Keywords = Hashimoto disease) OR Title or Keywords = Hashimoto's thyroiditis) OR Title or Keywords = chronic lymphocytic thyroiditis) OR Title or Keywords = Hashimoto thyroiditis) AND (((Title or Keywords = depression OR Title or Keywords = anxiety) OR Title or Keywords = depressive symptoms) OR Title or Keywords = depressive mood)) | 24 |
