# Supplementary material for: Association between depression and anxiety disorders with euthyroid Hashimoto's thyroiditis: A systematic review and meta-analysis
Source: Compr Psychoneuroendocrinol. 2024 Dec 2;20:100279. doi: 10.1016/j.cpnec.2024.100279 (PMC11665666; doi:10.1016/j.cpnec.2024.100279)
Supplement: Multimedia component 3 [file mmc3.docx]

**Table S3**. Demographic and Clinical Characteristics of Included Studies

| **Outcome** | **Studies** | **No. of Patients** | **WMD /SMD or OR** | **95% CI** | ***p*-value** | **Heterogeneity** | | | |
| --- | --- | --- | --- | --- | --- | --- | --- | --- | --- |
|  |  | **HT/Normal** |  |  |  | Chi^2^ | df | *P*-value | *I^2^* (%) |
| Age (years) | 9 | 517/520 | 1.61 | [-0.43-3.65] | 0.12 | 31.56 | 8 | 0.0001 | 75 |
| Gender (male) | 9 | 517/520 | 0.84 | [0.31-2.26] | 0.73 | 21.98 | 5 | 0.0005 | 77 |
| BMI (kg/m2) | 3 | 81/132 | 0.92 | [-0.00-1.84] | 0.05 | 1.14 | 2 | 0.57 | 0 |
| Waist circumference (cm) | 2 | 40/91 | 0.60 | [-3.86-5.06] | 0.79 | 0.69 | 1 | 0.40 | 0 |
| Marital status（married） | 5 | 285/363 | 1.20 | [0.82-1.76] | 0.35 | 3.05 | 4 | 0.55 | 0 |
| Education (>high school） | 6 | 301/381 | 0.89 | [0.50-1.57] | 0.68 | 13.11 | 5 | 0.02 | 62 |
| Work（yes） | 3 | 169/248 | 0.89 | [0.53-1.49] | 0.66 | 0.21 | 2 | 0.90 | 0 |
| TSH (µIU/mL) | 8 | 456/442 | 0.42 | [0.19-0.65] | 0.0003^a^ | 20.53 | 7 | 0.005 | 66 |
| Total T3 | 2 | 63/58 | -0.07 | [-0.16-0.01] | 0.09 | 0.24 | 1 | 0.62 | 0 |
| Free T3 | 4 | 226/240 | 0.12 | [-0.12-0.37] | 0.33 | 4.82 | 3 | 0.19 | 38 |
| Total T4 | 2 | 63/58 | 2.48 | [-3.57-8.54] | 0.42 | 1.04 | 1 | 0.31 | 4 |
| Free T4 | 8 | 425/420 | -0.08 | [-0.30-0.13] | 0.45 | 15.81 | 7 | 0.03 | 56 |
| Anti-TPO (IU/mL) | 8 | 379/429 | 2.46 | [1.61-3.31] | <0.00001^a^ | 162.72 | 7 | <0.00001 | 96 |
| Anti-Tg (IU/mL) | 5 | 313/326 | 1.50 | [0.93-2.07] | <0.00001^a^ | 37.26 | 4 | <0.00001 | 89 |

^a^ Statistically significant.

BMI, body mass index; WMD, weighted mean difference; SMD, standardized mean difference；OR, odds ratio; CI, confidence interval.
